# Residue L193P Mutant of RpoS Affects Its Activity During Biofilm Formation in *Salmonella* Pullorum

**DOI:** 10.3389/fvets.2020.571361

**Published:** 2020-11-05

**Authors:** Zheng Feng, Muhanad El Hag, Tao Qin, Yinping Du, Sujuan Chen, Daxin Peng

**Affiliations:** ^1^College of Veterinary Medicine, Yangzhou University, Yangzhou, China; ^2^Jiangsu Co-Innovation Center for the Prevention and Control of Important Animal Infectious Diseases and Zoonoses, Yangzhou, China; ^3^Jiangsu Research Centre of Engineering and Technology for Prevention and Control of Poultry Disease, Yangzhou, China; ^4^Joint Laboratory Safety of International Cooperation of Agriculture and Agricultural-Products, Yangzhou University, Yangzhou, China

**Keywords:** *Salmonella* pullorum, rpoS, biofilm, promoter, mutation

## Abstract

The role of alternative sigma factor RpoS in regulating biofilm formation may differ in various *Salmonella* Pullorum strains. In this study, the biofilm-forming ability of two *Salmonella* Pullorum strains S6702 and S11923-3 were compared. The biofilm forming ability of S11923-3 was much stronger than that of S6702. After knocking out the *rpoS* gene, S11923-3Δ*rpoS* had significantly reduced biofilm while S6702Δ*rpoS* demonstrated similar biofilm compared with each parent strain. The analysis of RpoS sequences indicated two amino acid substitutions (L193P and R293C) between S6702 and S11923-3 RpoS. A complementation study confirmed that the expression of S11923-3 RpoS rather than S6702 RpoS could restore the biofilm-forming ability of Δ*rpoS* strains and the L193P mutation contributed to the restoration of the biofilm-forming ability. Further study indicated that RpoS with the L193P mutant had significantly improved expression level and binding activity to RNAP and *csgD* gene promoter, which increased the efficacy of the *csgD* gene promoter and biofilm-forming ability. Therefore, the L193P mutation of RpoS is critical for stronger biofilm formation of *Salmonella* Pullorum.

## Introduction

Bacteria have evolved various cellular stress responses to survive in different environments. Using alternative sigma (σ) subunits of RNA polymerase (RNAP) that consists of five principal subunits (β, β', ω, and two α subunits) to directly initiate transcription of different classes of promoters is a major strategy employed by bacteria to modify the expression of genes (1, 2). The σ^S/38^ (RpoS), as an alternative sigma factor, is a central regulator enabling many Gram-negative bacteria to adapt to stress conditions and specialized environments (3, 4). RpoS can also play important roles in biofilm formation by regulating the central regulator CsgD (5).

Curli fimbriae and cellulose are two important components of biofilms. As a central regulator in the *Salmonella* biofilm-forming pathway, CsgD controlled by RpoS regulates the expression of the curli fimbriae *csgBAC* operon and putative transmembrane protein AdrA. AdrA is involved in regulating the production of cellulose by activating *basABZC-bcsEFG* operons (5, 6).

Pullorum disease caused by *Salmonella* Pullorum is a vertical or horizontal transmission disease that causes serious losses in the poultry industry (7). *S*. Pullorum can form biofilms, and different strains' biofilm-forming abilities vary (8, 9). Bacteria form biofilms to resist antimicrobials (10), host defense (11), desiccation, and disinfectants (12). Many studies showed that biofilms cause the majority of chronic infections and make them difficult to eradicate (13). In a previous study, we found that *S*. Pullorum strain S6702 could form biofilm, but deletion of the *rpoS* gene in S6702 had no effects on its biofilm formation (14). In the present study, we identified a stronger *rpoS*-dependent biofilm producer *S*. Pullorum strain S11923-3 and compared the role of RpoS in biofilm formation between the two *S*. Pullorum strains.

## Materials and Methods

### Bacterial Strains, Plasmids, and Growth Conditions

The bacterial strains and plasmids used in this study are listed in Table 1. All of the mutants were derived from *S*. Pullorum S6702 (14) and S11923-3 (this study). The strains were routinely cultured in Luria-Bertani (LB, Oxoid) broth and LB agar medium containing 1.5% (w/v) agar with appropriate antibiotics at the following concentrations: 20 μg/mL^−1^ of chloramphenicol and 100 μg/mL^−1^ of ampicillin. Tryptic soy broth diluted 1:10 (1/10 TSB, BD) in distilled water was used for the biofilm assays.

**Table 1:** Bacterial strains and plasmids used in this study.

**Strains or plasmids**	**Description**	**References**
**Strains**		
***S*****. Pullorum**		
S6702	Wild type	(14)
S6702Δ*rpoS*	S6702 without *rpoS* gene	(14)
S11923-3	Wild type	This study
S11923-3Δ*rpoS*	S11923-3 without *rpoS* gene	This study
S0219-S1228 (12 strains)	*S*. Pullorum isolates	This study
S6702Δ*rpoS*R7	S6702Δ*rpoS* containing pGEX-6P-1-7S	This study
S6702Δ*rpoS*R7B	S6702Δ*rpoS* containing pGEX-6P-1-7B	This study
S6702Δ*rpoS*Rp7	S6702Δ*rpoS* containing pGEX-6P-1-p7	This study
S6702Δ*rpoS*R9	S6702Δ*rpoS* containing pGEX-6P-1-9S	This study
S6702Δ*rpoS*R9B	S6702Δ*rpoS* containing pGEX-6P-1-9B	This study
S6702Δ*rpoS*Rp9	S6702Δ*rpoS* containing pGEX-6P-1-p9	This study
S6702-9rpoS	S6702 substitute with S11923-3 rpoS	This study
S6702-9rpoSB	S6702 substitute with S11923-3 rpoS containing residue P193L mutation	This study
S11923-3Δ*rpoS*R7	S11923-3Δ*rpoS* containing pGEX-6P-1-7S	This study
S11923-3Δ*rpoS*R7B	S11923-3Δ*rpoS* containing pGEX-6P-1-7B	This study
S11923-3Δ*rpoS*Rp7	S11923-3Δ*rpoS* containing pGEX-6P-1-p7	This study
S11923-3Δ*rpoS*R9	S11923-3Δ*rpoS* containing pGEX-6P-1-9S	This study
S11923-3Δ*rpoS*R9B	S11923-3Δ*rpoS* containing pGEX-6P-1-9B	This study
S11923-3Δ*rpoS*Rp9	S11923-3Δ*rpoS* containing pGEX-6P-1-p9	This study
S11923-3-7rpoS	S11923-3 substitute with S6702 rpoS	This study
S11923-3-7rpoSB	S11923-3 substitute with S6702 rpoS containing residue L193P mutation	This study
***E. coli***		
Trans1T1	F^−^ϕ80(*lac*Z)ΔM15Δ*lac*X74*hsd*R(r${}_{\text{k}}^{-}$,m${}_{\text{k}}^{+}$)Δ*rec*A1398*end*A1*ton*A	Transgen
BL21(DE3)	F^−^ompT hsdS(r${}_{\text{B}}^{-}$m${}_{\text{B}}^{+}$)gal dem(DE3)	Transgen
**Plasmids**		
pKD46	Lambda red helper plasmid expressing homologous recombinase	(15)
pKD3	Plasmid knockout vector Cm^R^	(15)
pCP20	Plasmid knockout vector Amp^R^ & Cm^R^	(15)
pUC18	Cloning vector Amp^R^	Novagen
pSV-β-gal	Expression vector Amp^R^	Novagen
pcsgD-lacZ	pUC18 containing the promotor of *csgD* and *lacZ* gene	This study
pET32a	Expression vector Amp^R^	Novagen
pET32a-7S	pET32a containing *rpoS* from S6702	This study
pET32a-9S	pET32a containing *rpoS* from S11923-3	This study
pGEX-6P-1	Expression vector Amp^R^	Novagen
pGEX-6P-1-7S	pGEX-6P-1 containing *rpoS* from S6702	This study
pGEX-6P-1-7B	pGEX-6P-1 containing *rpoS* from S6702 with changed residue 193 Pro	This study
pGEX-6P-1-p7	pGEX-6P-1 containing *rpoS* and its promotor from S6702 without Tac promotor	This study
pGEX-6P-1-9S	pGEX-6P-1 containing *rpoS* from S11923-3	This study
pGEX-6P-1-9B	pGEX-6P-1 containing *rpoS* from S11923-3 with changed residue 193 Leu	This study
pGEX-6P-1-p9	pGEX-6P-1 containing *rpoS* and its promotor from S11923-3 without Tac promotor	This study

### Construction of Mutant and Complementary Strains

The *rpoS* genes of *S*. Pullorum strains S6702 and S11923-3 were deleted using lambda red-mediated mutagenesis procedures (16). All of the mutants were verified by PCR amplification using the primer pair rpoS-F/R. The PCR products were confirmed by DNA sequencing (TsingKe Biological Technology Company, China).

To construct the complementary strains, the *rpoS* genes were amplified using primer pair rpoS-HF/HR and chromosomal DNA from the S6702 and S11923-3 strains as templates. The PCR products were digested by restriction endonuclease *Bam*HI and *Xho*I and cloned into pGEX-6P-1 to obtain pGEX-6P-1-7S and pGEX-6P-1-9S plasmids. The pGEX-6P-1-7B and pGEX-6P-1-9B plasmids with residue 193 mutants in RpoS were constructed using a Mut Express MultiS Fast Mutagenesis Kit V2 (Vazyme) based on the pGEX-6P-1-7S and pGEX-6P-1-9S plasmids. The pGEX-6P-1-p7 and pGEX-6P-1-p9 plasmids containing RpoS promotor without Tac promotor were constructed using a ClonExpress II One Step Cloning Kit (Vazyme) with primers listed in Supplementary Table 1. All of the plasmids (pGEX-6P-1-7S, pGEX-6P-1-7B, pGEX-6P-1-p7, pGEX-6P-1-9S, pGEX-6P-1-9B, and pGEX-6P-1-p9) were transformed into *S*. Pullorum S6702Δ*rpoS* and S11923-3Δ*rpoS* by electroporation to produce complementary strains S6702Δ*rpoS*R7, S6702 Δ*rpoS*R7B, S6702Δ*rpoS*R9, S6702Δ*rpoS*R9B, S6702Δ*rpoS*Rp7, S6702Δ*rpoS*Rp9, S11923-3Δ*rpoS*R7, S11923-3Δ*rpoS*R7B, S11923-3Δ*rpoS*R9, S11923-3Δ*rpoS*R9B, S11923-3Δ*rpoS*Rp7, and S11923-3Δ*rpoS*Rp9.

To construct the *rpoS* substitution strains, the *rpoS* genes with residues 193/293 substitution were amplified using primer pair rpoS-F1/R1 (Supplementary Table 1), the *cat* gene was amplified from plasmid pKD3 using primer pair cat-F/R, the *rpoS-cat* genes were amplified using overlapped *rpoS* and *cat* gene PCR products as template and rpoS-cat-F/R as primer pair. The *rpoS-cat* PCR products with DNA homology to the DNA regions flanking *rpoS* gene were transformed into S6702 or S11923-3 competent cells carrying lambda red helper plasmid pKD46. Transformants were selected on LB plates containing 20 μg/mL^−1^ of chloramphenicol. The mutant strains were verified by PCR amplification and DNA sequencing (TsingKe Biological Technology Company, China), and named as S6702-9rpoS, S6702-9rpoSB, S11923-3-7rpoS and S11923-3-7rpoSB (Table 2).

**Table 2:** Amino acid substitutions of RpoS and crystal violet staining quantification in *S. Pullorum* strains.

**Strains**	**Residue 193**	**Residue 293**	**OD_**550**_**
S6702	CTG(Leu)	CGT(Arg)	0.52 ± 0.04
S11923-3	CCG(Pro)	TGT(Cys)	1.92 ± 0.28
S0219	CCG(Pro)	TGT(Cys)	1.60 ± 0.01
S0711	CCG(Pro)	CGT(Arg)	1.42 ± 0.09
S0226	CCG(Pro)	TGT(Cys)	1.48 ± 0.07
S1219	CCG(Pro)	CGT(Arg)	1.46 ± 0.12
S0825	CCG(Pro)	CGT(Arg)	0.80 ± 0.10
S1129	CCG(Pro)	CGT(Arg)	1.12 ± 0.02
S0120	CCG(Pro)	CGT(Arg)	1.14 ± 0.09
S0227	CCG(Pro)	TGT(Cys)	1.65 ± 0.16
S0303	CCG(Pro)	TGT(Cys)	1.64 ± 0.04
S1223	CCG(Pro)	CGT(Arg)	1.74 ± 0.24
S1217	CCG(Pro)	CGT(Arg)	1.58 ± 0.11
S1228	CCG(Pro)	CGT(Arg)	1.34 ± 0.30
S6702-9rpoS	CCG(Pro)	TGT(Cys)	0.98 ± 0.17
S6702-9rpoSB	CTG(Leu)	TGT(Cys)	0.51 ± 0.08
S11923-3-7rpoS	CTG(Leu)	CGT(Arg)	1.18 ± 0.18
S11923-3-7rpoSB	CCG(Pro)	CGT(Arg)	1.89 ± 0.41

### Biofilm Assays

Biofilm formation ability was measured as previously described (14, 17). Overnight broth cultures of each strain were diluted at 1:100 in 1/10 TSB and 100 μL of each bacterial suspension was added to 96-well U-bottomed plates (Corning). The plates were incubated at 28°C for 24 h without shaking. The supernatant was discarded and the wells were gently washed three times with 200 μL of distilled water to remove non-adherent bacteria. A total of 100 μL of 0.4% crystal violet was then added for 20 min. The wells were washed three times with distilled water. The remaining crystal violet bound to the adherent cells was solubilized with 100 μL of 25% acetone with anhydrous ethanol and the optical density (OD_550_) was measured in Microplate Reader (Bio-Rad). The assays were conducted three times using duplicate wells in each independent assay.

Curli fimbriae and cellulose production was evaluated by colony morphology in Congo red plates with 40 μg/mL of Congo red (Sangon Biotech) and 20 μg/mL of Coomassie brilliant blue (Sangon Biotech) and LB agar plates with 200 μg/mL of calcofluor (Sigma-Aldrich) after the inoculation plates were incubated at 28°C for 4 days (18). The colonies' fluorescence was observed under UV light.

### Field Emission Scanning Electron Microscopy

The biofilm morphology was also determined using scanning electron microscopy as previously described (14). Polystyrene coverslips (d = 14 mm) were inoculated with diluted overnight broth cultures and incubated at 28°C for 24 h so biofilm would form on the coverslips. Then the coverslips were washed three times with 0.1 M of phosphate-buffered saline (PBS, pH 7.0) and fixed in 3% glutaraldehyde in PBS for 2 h at 4°C. Afterward, the coverslips were washed three times with PBS and dehydrated with increasing concentrations of ethanol. The samples were dried using critical point-drying for 5 h, coated with gold palladium alloy, and observed with a Gemini 300 SEM (Carl Zeiss).

### Quantitative Real-Time PCR (qRT-PCR) Analysis

Bacteria were grown in 1/10 TSB medium at 28°C for 24 h in 60 mm dishes (Corning). The supernatant was discarded and the bacteria accumulated in the biofilms under the dishes were scraped. The total RNA was extracted using a Bacterial RNA Kit (Omega). The cDNA was synthesized using a PrimeScript RT reagent Kit with gDNA Eraser (Takara) and quantified via TB Green Premix Ex Taq (Takara). The gene transcript levels were tested in triplicate for real-time PCR in a Linegene 9600 Plus machine (Bioer). Primer pairs of Q-gyrB-F/R, Q-csgD-F/R, Q-csgA-F/R, and Q-bcsA-F/R (Supplementary Table 1) were used for the mRNA detection of *gyrB, csgD, csgA*, and *bcsA*, respectively. The target genes' mRNA levels were normalized to the *gyrB* mRNA levels (2^−ΔΔCt^) (19–21).

### Immunoblotting Analyses

Bacteria were grown in 1/10 TSB in 60 mm dishes at 28°C for 8 and 24 h to test the protein expression. After the dishes were washed with PBS, the bacterial cells were collected and quantified to OD_600_ = 1.0, then suspended in SDS sample buffer (Beyotime Biotechnology). The samples were resolved in 12% SDS polyacrylamide gels, transferred to PVDF membranes (Millipore), and analyzed by immunoblotting using anti-RpoS (NeoClone) and anti-RpoA (NeoClone) antibodies. Bands were developed using anti-mouse-HRP (Abcam) and an ECL detection system (Tanon).

### Protein Expression and Purification

The *rpoS* genes were amplified using rpoS-yF/yR as primers and chromosomal DNA from the S6702 and S11923-3 as templates. The PCR products were inserted between the *Bam*HI and *Xho*I sites of the pET32a, which were called pET32a-7S and pET32a-9S, respectively. The plasmids were transformed to *Escherichia coli* BL21 (DE3) for the overexpression and purification of recombinant RpoS. Bacterial cells were grown at 37°C until OD_600_ of 0.4–0.6 and 1 mM of IPTG was added. The cells were then grown at 16°C for 12 h and harvested by centrifugation at 5,000 g for 10 min. His-tagged proteins were purified by incubation with Ni-NTA resin (Genscript). The resin was washed with at least 8 column volumes (CV) of wash buffer (300 mM of NaCl, 50 mM of Na_2_HPO_4_ with a pH of 8, and 10 mM of imidazole). The protein was eluted with elution buffer (300 mM of NaCl, 250 mM of Na_2_HPO_4_ with a pH of 8, and 250 mM of imidazole) in 1 CV fractions. The fractions were analyzed by SDS-PAGE and the purified RpoS was prepared for binding assays and electrophoretic mobility shift assays (EMSAs).

### Binding Assay

RNAP-clarified lysates were prepared via the following steps: 50 mL of each bacterial culture incubated in TSB at 37°C overnight was pelleted at 5,000 g for 10 min. The pellets were resuspended in 8 mL pull-down buffer (140 mM of NaCl, 6.5 mM of sodium-phosphate with a pH of 7.4, and 0.02% Tween-20) and then sonicated on ice for up to 15 min at 1 min intervals. Cell debris was cleared by centrifugation at 14,000 rpm for 30 min at 4°C, and the supernatant was passed through a 0.45 μm filter. The clarified lysate protein concentration was measured by Bradford assays (Thermo Fisher Scientific).

His-tag Dynabeads (50 μL, Invitrogen) were incubated with 700 μL of purified RpoS protein (2.0 mg/mL) and rotated at 25 rpm for at least 20 min at room temperature. The beads were washed according to the manufacturer's protocols with 300 μL wash buffer (600 mM of NaCl, 100 mM of sodium-phosphate with a pH of 8, and 0.02% Tween-20). Then the clarified lysates were incubated with the beads for 15 min and washed four times. The samples were then eluted from the beads by incubation in 100 μL of elution buffer (300 mM of NaCl, 50 mM of sodium-phosphate with a pH of 8, 300 mM of imidazole, and 0.01% Tween-20). The samples were resolved in SDS-PAGE, transferred onto PVDF membranes (Millipore), and probed with primary anti-RpoS, anti-RpoA, and anti-RpoB antibodies (22, 23) and secondary antibody goat anti-mouse-HRP (Abcam). Images were captured using the Tanon Imaging System (Tanon).

### Electrophoretic Mobility Shift Assays (EMSAs)

EMSAs were conducted as previously described (24). Briefly, a 646-bp *csgD* gene promoter including 237 nucleotides in the open reading frame was amplified from S11923-3 and purified with an AxyPrep DNA Gel Extraction Kit (Axygen), and the purified products were PCR labeled using FAM-modified primer. The binding reaction was conducted with non-specific competitor DNA (poly dI-dC, Sigma-Aldrich) in buffer (pH 7.4) containing 750 mM of NaCl, 0.5 mM of DTT, 0.5 mM of EDTA, and 50 mM of Tris at 25°C for 30 min. A total of 20 μL of each reaction system contained 4 μL 5 × binding buffer (Beyotime Biotechnology) and 200 ng of poly dI-dC (Sigma-Aldrich). The final mixtures including DNA fragments and RpoS protein were run on a 6% SDS-PAGE. Then the images were scanned and observed using a fluorescence imaging system (Typhoon FLA 9500, GE Healthcare).

### β-Galactosidase Activity Assays

The promoters of *csgD* genes from strains S6702 and S11923-3 were amplified using primer pair csgD-F/R and the PCR products were inserted into pUC18 with *Kpn*I and *Bam*HI digestion to form pcsgD. The *lacZ* gene from plasmid pSV-β-gal was amplified using primer pair lacZ-F/R and the PCR products were inserted into pcsgD with *Bam*HI and *Pst*I digestion to form pcsgD-lacZ, in which the *lacZ* gene was downstream from the *csgD* gene promoter. The pcsgD-lacZ was transformed into S6702, S11923-3, and their *rpoS* deletion mutants. The bacteria harboring the plasmids were cultured in dishes at 28°C for 24 and 48 h without shaking. After collecting and measuring the absorbance at 600 nm, the samples were mixed with reaction buffer (60 mM of Na_2_HPO_4_, 40 mM of NaH_2_PO_4_, 10 mM of KCl, 1 mM of MgCl_2_, and 0.4 mg/mL of ONPG) and stop buffer (1 M of Na_2_CO_3_). The absorbance was measured at 420 nm. The β-galactosidase activity (Miller units) was calculated as previously described (25).

### Statistical Analysis

GraphPad Prism 6 was used for graph plotting and statistical analysis. All of the data are expressed as mean and standard deviations (SD). All of the statistical analyses were assessed using the two-tailed *t*-test. *P* < 0.05 were considered significant.

## Results

### RpoS Had Different Effects on Biofilm-Forming Ability in *Salmonella* Pullorum S6702 and S11923-3

**Figure 1:**
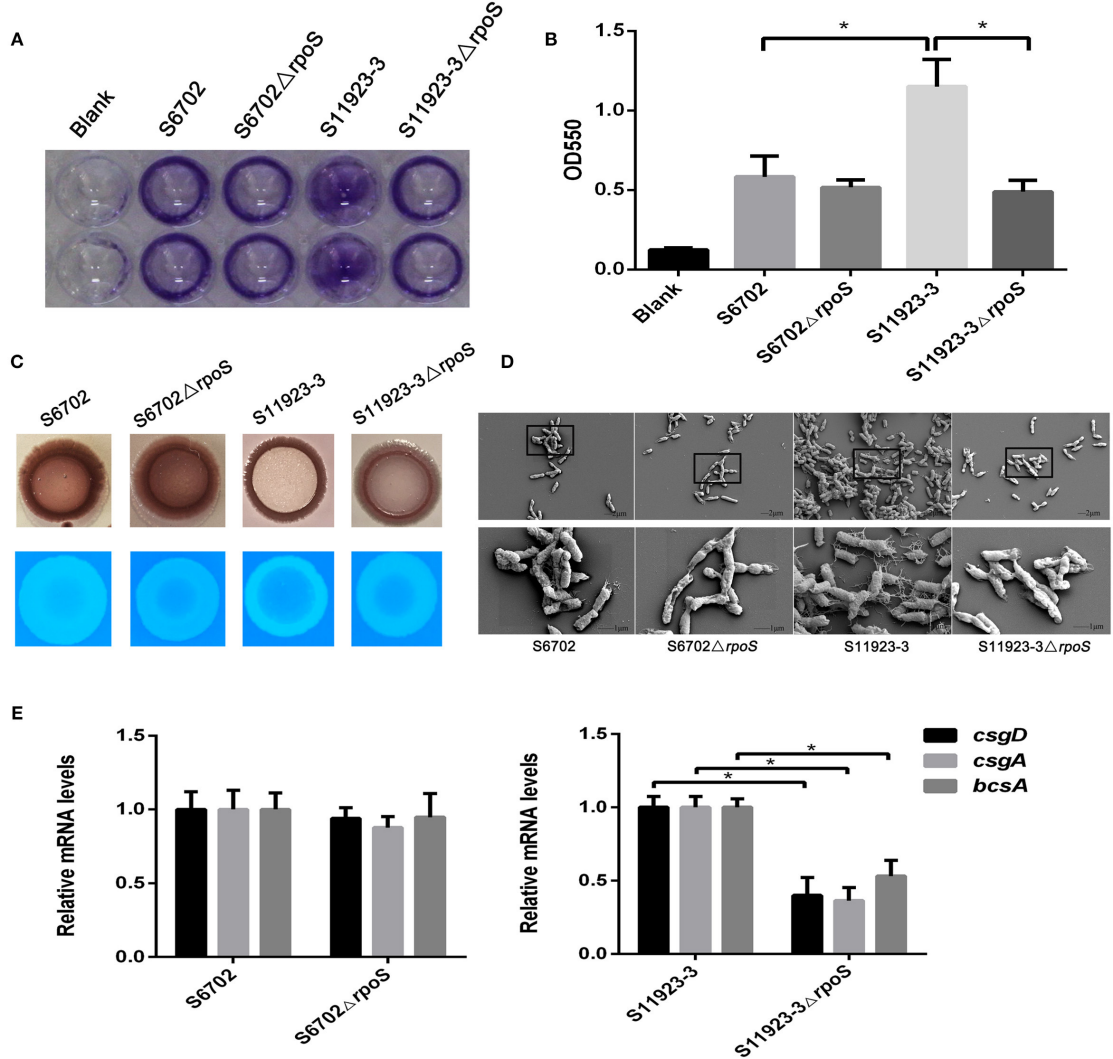
Effect of RpoS on biofilm formation in *Salmonella* Pullorum S6702 and S11923-3. **(A)** Crystal violet staining of bacteria grown in 96-well plates. Duplicate wells were used in each independent assay. **(B)** Quantification of crystal violet staining by measuring the optical density (OD_550_). Means and standard deviations from three independent experiments are shown. **(C)** Morphology of colonies after growth on Congo red and calcofluor agar plates. **(D)** Field emission scanning electron microscope observation of bacteria. The bottom figure (bars = 1 μm) originated from the black boxes in the top figure (bar = 2 μm). **(E)** The mRNA levels of *csgD, csgA*, and *bcsA* genes determined by qRT-PCR. The mRNA level of each gene was normalized by the mRNA level of the *gyrB* gene. The bars represent the means of three independent assays. *P < 0.05.

To explore the effects of RpoS on the biofilm formation of *S*. Pullorum, we constructed *rpoS* gene deletion mutants S6702Δ*rpoS* and S11923-3Δ*rpoS*. The biofilm-forming ability of strains S6702 and S11923-3 and their *rpoS* deletion mutants were determined. Crystal violet staining of bacterial strains on polystyrene plates showed that wild-type (WT) strain S6702 and its mutant strain S6702Δ*rpoS* had similar circle staining in the plate well walls (Figure 1A). The WT strain S11923-3 formed spot staining covering the wall and bottom of the plate well, while its mutant strain S11923-3Δ*rpoS* formed circle staining. After quantifying the crystal violet staining, the OD_550_ value of S11923-3 was significantly higher than that of S6702. The OD_550_ value of S11923-3Δ*rpoS* was significantly lower than that of S11923-3 and similar to that of S6702Δ*rpoS*, while S6702ΔrpoS had similar OD_550_ values as parent strain S6702 (Figure 1B).

Colony morphologies on the Congo red plates showed that WT strain S11923-3 appeared as a red, dry, and rough colony (rdar) after incubation at 28°C for 4 days, while the S11923-3Δ*rpoS* produced a pink and smooth colony (Figure 1C). However, S6702 and S6702Δ*rpoS* produced an rdar colony. In calcofluor staining assays, the S11923-3Δ*rpoS* strain exhibited reduced fluorescence compared with the parent strain, while S6702 and S6702Δ*rpoS* showed similar fluorescence (Figure 1C).

Field emission scanning electron microscopy analysis of the biofilm formation showed that the WT strain S11923-3 had clusters of bacterial cells and meshwork-like structures surrounding the bacterial surface, while the WT strain S6702 and mutant strains S6702Δ*rpoS* and S11923-3Δ*rpoS* had a small number of bacterial cells and tiny fiber structures surrounding the bacterial surface (Figure 1D).

The transcriptional levels of *csgD, csgA*, and *bcsA* genes related to biofilm formation were determined by relative qRT-PCR. The results showed that the three genes' transcriptional levels were significantly reduced in S11923-3Δ*rpoS* compared with the parent strain (Figure 1E). The three genes' transcriptional levels were almost the same in strains S6702 and S6702Δ*rpoS*.

### Two Amino Acid Substitutions (L193P and R293C) Were Identified in the S6702 and S11923-3 RpoS Sequences

To investigate the differences in RpoS in the two strains, we compared the amino acid sequences of RpoS in strains S6702 and S11923-3. Only two amino acid substitutions of L193P and R293C were found in the S6702 and S11923-3 RpoS (Supplementary Figure 1). To detect the distribution of the two residues in *S*. Pullorum, another 12 *S*. Pullorum strains isolated from 2015 to 2017 in Jiangsu were randomly selected and their RpoS sequences were analyzed (Table 2). All of the *S*. Pullorum strains had Pro in residue 193 of the RpoS except for S6702, while the *S*. Pullorum strains had Arg (9/14) or Cys (5/14) in RpoS residue 293.

To verify effects of residues 193 and 293 of RpoS on biofilm formation, all *S*. Pullorum strains were subjected to polystyrene plate culture and crystal violet staining (Table 2). The OD_550_ value of S6702 with 193L and 293R pattern was significantly lower than that of other strains with 193P and 293R/C pattern, while the OD_550_ values of *S*. Pullorum strains with 193P and 293R pattern varied from 0.80 to 1.74 and the OD_550_ values of *S*. Pullorum strains with 193P and 293C pattern varied from 1.48 to 1.92, indicating that residue 193 of RpoS might make more contribution to biofilm formation.

### RpoS Residue 193 Was Critical for Biofilm Formation in *S*. Pullorum

**Figure 2:**
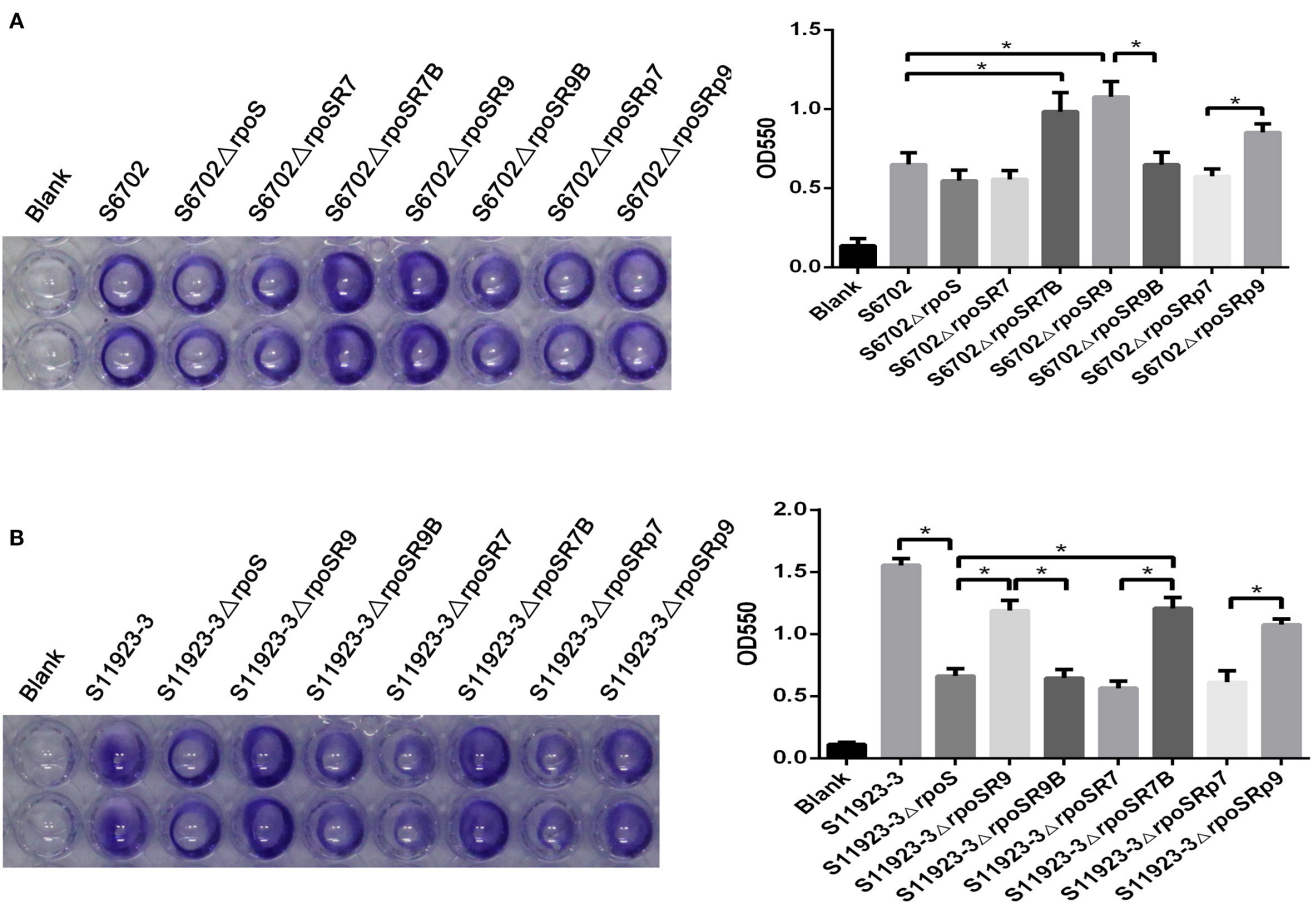
Determination of biofilm formation of S6702Δ*rpoS* **(A)** and S11923-3Δ*rpoS* **(B)** complemented with two point mutants in residues 193 and 293 of RpoS. All of the strains were cultured in TSB medium first. After overnight cultivation, cultures were diluted in 1/10 TSB and grown in 96-well plates (100 μL/ well) at 28°C for 24 h without shaking. Discarding the supernatant and washing the wells gently with distilled water to remove non-adherent bacteria. After staining with 0.4% crystal violet for 20 min, washed the wells and the remaining crystal violet was solubilized with 100 μL of 25% acetone with anhydrous ethanol. Crystal violet staining quantification was tested by measuring the optical density (OD_550_). Means and standard deviations from three independent experiments are shown. *P < 0.05.

To further ensure the residue effects of RpoS on biofilm formation in S6702 and S11923-3, *rpoS* complementary strains and substitution strains were constructed and determined by crystal violet staining test using the wild-type strains and *rpoS* mutant strains as contrast. Crystal violet staining of the biofilm formation in these strains showed that S6702Δ*rpoS*R7 complemented with S6702 RpoS demonstrated similar staining or OD_550_ values than S6702Δ*rpoS*, while S6702Δ*rpoS*R9 complemented with S11923-3 RpoS had stronger staining or higher OD_550_ values than S6702Δ*rpoS* and S6702 (Figure 2A). S11923-3Δ*rpoS*R7 complemented with S6702 RpoS showed similar staining or OD_550_ values as S11923-3Δ*rpoS*, while S11923-3Δ*rpoS*R9 complemented with S11923-3 RpoS recovered the staining and had higher OD_550_ values (Figure 2B). These data indicated the efficacy of S11923-3 RpoS in biofilm formation is higher than that of S6702 RpoS.

After exchanging residue 193 between S6702 RpoS and S11923-3 RpoS, the complementary strain S6702Δ*rpoS*R7B demonstrated significantly stronger staining or higher OD_550_ values than the mutant S6702Δ*rpoS* strain; in contrast, the complementary S6702Δ*rpoS*R9B strain had similar staining or OD_550_ values as the mutant strain S6702Δ*rpoS* (Figure 2A). The same result was also observed in S11923-3 and its related strains (Figure 2B).

Biofilm formation of the substitution strains were also determined by crystal violet staining test, the OD_550_ values of S6702-9rpoS (193P, 293C) were significantly increased when compared with that of S6702, while OD_550_ values of S6702-9rpoSB (193L, 293C) were significantly decreased when compared with that of S6702-9rpoS (Table 2). In contrast, the OD_550_ values of S11923-3-7rpoS (193L, 293R) were significantly decreased when compared with that of S11923-3, while OD_550_ values of S11923-3-7rpoSB (193P, 293R) were significantly increased when compared with that of S11923-3-7rpoS. These data indicated that RpoS residue 193 rather than residue 293 facilitated the biofilm-forming ability in these two strains.

### Residue L193P Contributed to the RpoS Expression During Bacterial Biofilm Formation

**Figure 3:**
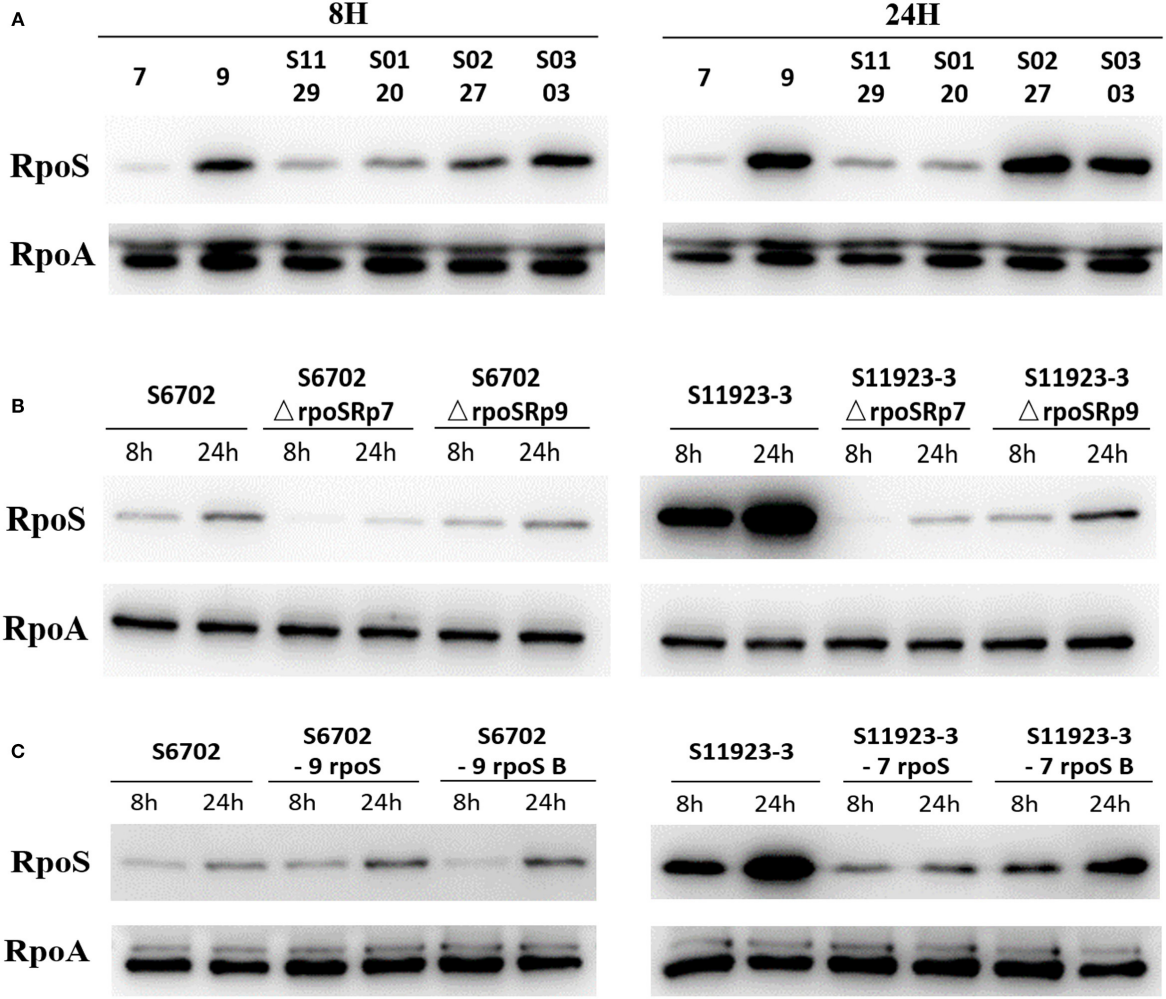
Determination of RpoS expression by immunoblotting analysis in different *S*. Pullorum isolates **(A)**, *rpoS* gene deletion mutants and complementary strains **(B)**, and *ropS* gene substitution strains **(C)** during biofilm formation. All of the strains were cultured in TSB medium first. Overnight cultures were diluted in 1/10 TSB medium in small dishes at 28°C without shaking for 8 and 24 h. Supernatant was discarded and the scraped samples were collected. The samples were quantified to OD_600_ = 1.0, then suspended in SDS sample buffer (Beyotime Biotechnology). The samples were resolved in 12% SDS polyacrylamide gels, transferred to PVDF membranes (Millipore), and analyzed by immunoblotting using anti-RpoS (NeoClone) and anti-RpoA (NeoClone) antibodies.

To determine the RpoS expression among different *S*. Pullorum strains, six strains were selected for determination of RpoS expression. Samples were collected after 8 h and 24 h of incubation and subjected to immunoblotting analysis. As shown in Figure 3A, the expression level of RpoS in strains S6702 with residues 193L and 293R pattern was the lowest at each time point, the expression levels of RpoS in strains S1129 and S0120 with residues 193P and 293R pattern were moderate and that of RpoS in strains S11923-3, S0227, and S0303 with residues 193P and 293C pattern were the highest, indicating that both residues 193 and 293 contribute to RpoS expression in *S*. Pullorum.

To test the RpoS expression level under the same conditions and eliminate the effects of plasmid promoter on the *rpoS* gene expression, the pGEX-6p-1-p7 and pGEX-6p-1-p9 plasmids with the substitution of Tac promoter by RpoS promoter of S6702 and S11923-3 were constructed and then transformed into S6702Δ*rpoS* and S11923-3Δ*rpoS*. The immunoblotting results showed that complement of S11923-3 RpoS had higher expression level than that of S6702 RpoS either in the mutant strains S6702Δ*rpoS* or S11923-3Δ*rpoS* (Figure 3B), indicating that residue L193P and R293C mutations could increase the RpoS expression significantly.

To verify the role of residues 193 and 293 on RpoS expression level, four *rpoS* gene substitution strains S6702-9rpoS, S6702-9rpoSB, S11923-3-7rpoS, and S11923-3-7rpoSB were constructed, and their RpoS expression levels were determined. As shown in Figure 3C, the RpoS expression level in S6702-9rpoS (193P, 293C) was higher than that in S6702 at each time point, and the RpoS expression level in S6702-9rpoSB (193L, 293C) was lower than that in S6702-9rpoS at 8 h incubation. In contract, the RpoS expression level in S11923-3-7rpoS (193L, 293R) was significantly lower than that in S11923-3 at each time point, and the RpoS expression level in S11923-3-7rpoSB (193P, 293R) was significantly higher than that in S11923-3-7rpoS. These data indicated that residue L193P increased the RpoS expression level.

### RpoS From the S11923-3 Strain Had Higher Activity to Initiate Biofilm Formation

**Figure 4:**
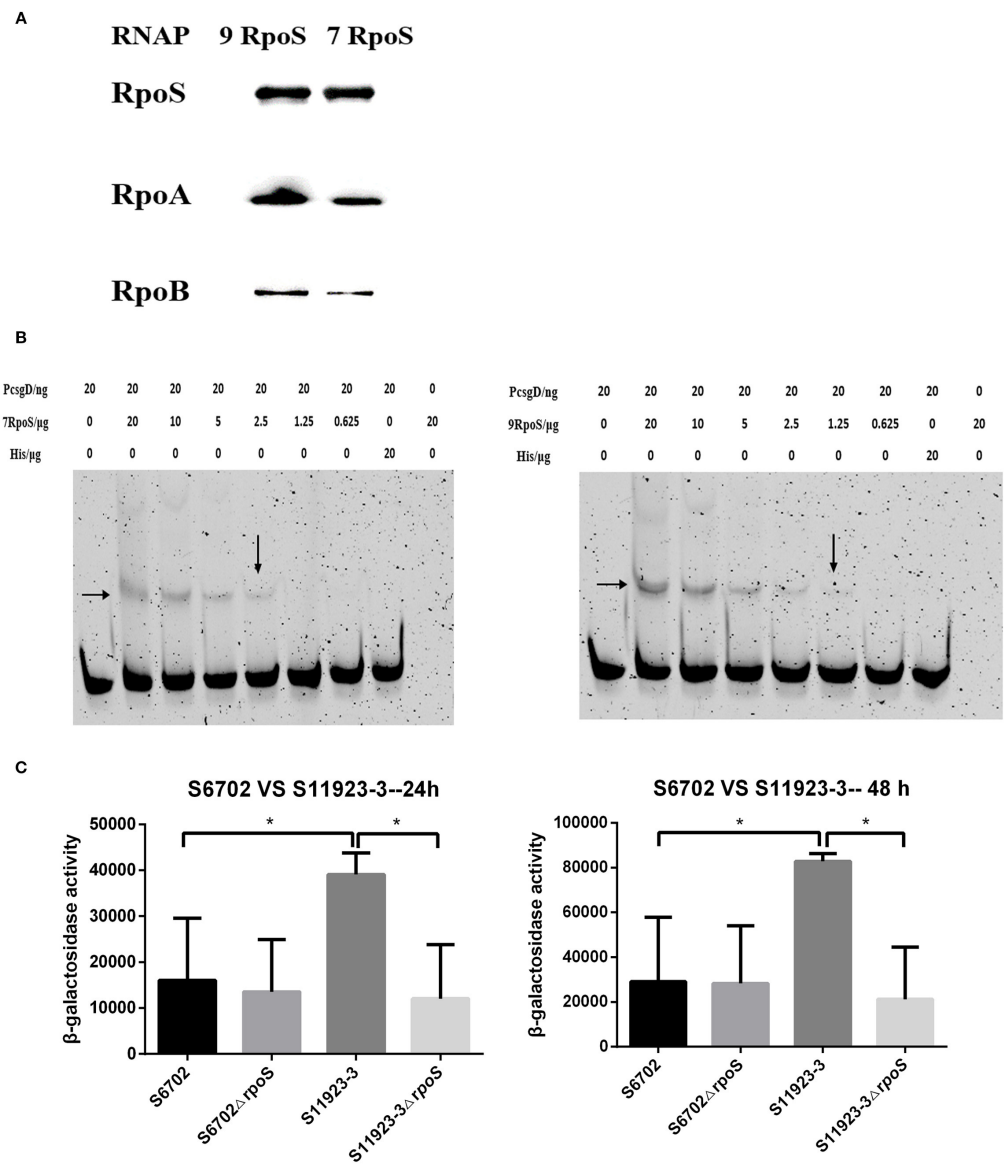
Determination of the binding ability of RpoS to RNAP and *csgD* promoter. **(A)** Immunoblotting analyses for binding activity of RpoS to RNAP. The purified RpoS protein (2.0 mg/mL) were incubated with his-tag Dynabeads (50 μL, Invitrogen). The beads were incubated with the clarified lysates including RNAP for 15 min. After being washed, the samples were resolved in SDS-PAGE, transferred onto PVDF membranes (Millipore), and probed with primary anti-RpoS, anti-RpoA, and anti-RpoB antibodies. **(B)** EMSA assays. The *csgD* promoter genes were PCR labeled using FAM-modified primer. The labeled DNA fragments and purified RpoS protein were incubated in the reaction system contained 4 μL 5 × binding buffer (Beyotime Biotechnology) and 200 ng of poly dI-dC (Sigma-Aldrich). The final mixtures were run on a 6% SDS-PAGE. The images were scanned and observed using a fluorescence imaging system (Typhoon FLA 9500, GE Healthcare). The data showed one representative experiment of three independent assays. **(C)** Determination of β-galactosidase activity. Plasmid pcsgD-lacZ was transformed into S6702, S11923-3, and their *rpoS* deletion mutants. The transformants were cultured in dishes at 28°C for 24 h and 48 h without shaking. After measuring the absorbance at 600 nm, the samples were mixed with reaction buffer and stop buffer. The absorbance was measured at 420 nm and the β-galactosidase activity was calculated. The bars represent the means of three independent assays. *P < 0.05.

To determine affinity of RpoS to RNAP, a pull-down assay with purified His-tagged RpoS (His-RpoS) and clarified lysates from mutant strains lacking RpoS was conducted. The RNAP pulled down by His-RpoS from the lysate was confirmed by immunoblotting analyses with anti-RpoS, anti-RpoA, and anti-RpoB antibodies (Figure 4A). When the RpoS was adjusted at the same concentration, the band intensities of RpoA and RpoB bound to S11923-3 RpoS were stronger than that bound to S6702 RpoS, indicating that S11923-3 RpoS had stronger binding capacity to RNAP.

CsgD plays an important role in regulating biofilm formation of *Salmonella* and its expression is controlled by RpoS (5). To test the recognition of RpoS to downstream *csgD* gene promoter, EMSA assays were conducted. As shown in Figure 4B, RpoS bound and shifted with DNA fragments in a dose-dependent manner, but S11923-3 RpoS bound a lower concentration (1.25 μg) of DNA probes than S6702 RpoS (2.5 μg), indicating that S11923-3 RpoS had stronger binding activity to *csgD* promoter.

To test the role of RpoS in regulating CsgD expression, a report plasmid was constructed. The results showed that β-galactosidase activity in the WT S6702 strain was lower than that in the WT S11923-3 strain after 24 h and 48 h of incubation (Figure 4C). β-galactosidase activity in the mutant S6702Δ*rpoS* strain was similar as that in S6702. However, β-galactosidase activity in S11923-3Δ*rpoS* was significantly lower than that in S11923-3 (Figure 4C). These data indicated that S11923-3 RpoS enhanced the expression activity of *csgD* promoter, and loss of the RpoS resulted in reduced expression activity of *csgD* promoter.

## Discussion

Gram-negative bacteria have many σ factors including RpoD, RpoS, RpoE, RpoH, and RpoN that bind with core RNAP (E) to form holoenzyme Eσ. Eσ enables specific binding to gene promoters and is required for transcription initiation (26). *S*. Typhimurium can form biofilms that are coordinated by a sophisticated network of signaling pathways through the expression of central biofilm regulator CsgD (5). However, the dependence of CsgD expression on RpoS may be variety. Römling proved that biofilm formation and the expression of central regulator CsgD is dependent on the alternative sigma factor RpoS (6). Further study found that the regulation of CsgD is partially independent of RpoS in *S*. Enteritidis (27). In a previous study, we found that *S*. Pullorum strain S6702 could form biofilm independent of sigma factor RpoS (14). In this study, we compared the role of RpoS in biofilm formation between *S*. Pullorum strains S6702 and S11923-3. And three methods were applied to determine the biofilm-forming ability of S6702, S11923-3, and their *rpoS* deletion mutants. All data confirmed that the biofilm-forming ability of S11923-3Δ*rpoS* was significantly reduced compared to that of parent strain S11923-3, while there were no changes in the biofilm formation in S6702Δ*rpoS*. The qRT-PCR results showed that the transcriptional levels of *csgD, csgA*, and *bcsA* genes related to biofilm formation also decreased significantly in strain S11923-3Δ*rpoS*. These data indicated that the biofilm-forming ability of S11923-3 is dependent on RpoS.

By comparing the RpoS sequences of S6702 and S11923-3, only two nucleotides (T578C and C877T) or two amino acid substitutions (L193P and R293C) were found. Then complementary strains and substitution strains with combinational substitution of residues 193 and 293 were constructed and their biofilm formation abilities were determined by crystal violet staining (Table 2 and Figure 2). The results indicated that residue L193P of RpoS could enhance the biofilm formation in *S*. Pullorum. It has been shown that accumulation of RpoS coincided with the expression of curli fimbriae and other morphological and physiological changes (28). In this study, S11923-3 RpoS (193P, 293C) had higher expression level than S6702 RpoS (193L, 293R) and residue L193P substitution increased the RpoS expression significantly while residue P193L substitution decreased the RpoS expression significantly (Figure 3C). According to Table 2 and Supplementary Figure 2, the biofilm formation ability was consistent with the RpoS expression in Figure 3C indicating that residue L193P of RpoS could increase the RpoS expression level further to enhance the biofilm formation in *S*. Pullorum.

Regulation of RpoS synthesis in *E. coli* is well-studied (29). Multiple levels including *rpoS* transcription, translation, and protein stability of regulation affect synthesis of RpoS. Transcription regulation is primarily from the *rpoS* promoter, embedded within the upstream *nlpD* gene contributing to basal level expression of RpoS in exponential phase (30, 31). 5′-UTR part of *rpoS* mRNA with relevant secondary structures and sRNA binding regions folds back to occlude ribosome entry and translation. sRNAs (for example, DsrA, ArcZ, RprA, etc.) can open the secondary structure, promoting translation (32–34). Synthesis of anti-adaptors like IraD enhances RssB to deliver RpoS to the clpPX protease for RpoS degradation (35, 36). Our study showed that there was no difference in *rpoS* gene promoter sequences between strains S6702 and S11923-3 (data not shown) and the transcript level of S11923-3 was higher than that of S6702 (Supplementary Figure 3). The mechanism for enhanced RpoS expression by L193P substitution need to be further studied.

RpoS combines with RNA polymerase (RNAP) to regulate many genes by binding to gene promotors and initiating transcription that enables bacteria to survive adverse conditions (37, 38). Research also showed that missense mutants in RpoS can affect its function including interacting with RNAP in *E. coli* (STEC) isolates. Substitution of Ile128 with Pro128 in *E. coli* abolishes RpoS activity including response to oxidative stresses and ability to bind RNAP (3, 39). Our study identified two amino acid substitutions in RpoS (L193P and R293C) and the binding assay results demonstrated that S11923-3 RpoS had stronger affinity for RNAP (Figure 4A). Amino acid substitutions of R141S and A157T in RpoS impair its ability to bind downstream promoters but do not affect its affinity to RNAP (40). Thus, we conducted EMSA assays to verify the promotor-binding ability of RpoS. The EMSA assays showed that RpoS could bind the *csgD* promoter DNA and S11923-3 RpoS had stronger ability to bind promoter DNA than S6702 RpoS (Figure 4B). In the reporter assay, S11923-3 had higher β-galactosidase activity than S6702, which demonstrated that S11923-3 RpoS induced more CsgD expression than S6702 RpoS (Figure 4C). In addition, when the *csgD* promoter sequences in these two strains were compared, no difference was found in the two promoters (data not shown), which removed the impact of the *csgD* promoter. Overall, the results of RpoS activities indicated that S11923-3 RpoS (193P) had stronger effects on biofilm formation in *S*. Pullorum by enhancing the affinity to RNAP and activating *csgD* promotor.

The cellular concentration of RpoD molecules exceeds that of core RNAP, suggesting that σ factors compete for binding to a limited number of core RNAP (40, 41). Both RpoD and RpoS can interact with RNA polymerase and regulate *csgBA* promoter expression in *Salmonella* (5). RpoE is another sigma factor that can affect biofilm formation in *S*. Pullorum (42). In other bacteria such as *Pseudomonas putida*, RpoD can mediate biofilm formation at a low level when RpoS is absent (43). In our study, we found that the biofilm-forming ability of *S*. Pullorum S6702 was RpoS-independent, which might be because other σ factors maintain a low level of biofilm formation after deleting RpoS. In strain S11923-3, other σ factors cannot initiate high levels of biofilm formation after deleting RpoS, showing a RpoS-dependent biofilm formation characteristic.

In conclusion, we found a new potential amino acid site (residue 193P) in RpoS that can enhance the RpoS expression level, binding activity to RNAP and expression activity of *csgD* gene promoter, resulting in enhanced biofilm formation in *S*. Pullorum.

## Data Availability Statement

The raw data supporting the conclusions of this article will be made available by the authors, without undue reservation.

## Author Contributions

ZF and DP conceived this study. ZF and MH conducted the research. ZF, TQ, and DP analyzed the data. ZF, YD, SC, and DP wrote the paper. All authors contributed to the article and approved the submitted version.

## Conflict of Interest

The authors declare that the research was conducted in the absence of any commercial or financial relationships that could be construed as a potential conflict of interest.
