# Supplementary material for: Residue L193P Mutant of RpoS Affects Its Activity During Biofilm Formation in Salmonella Pullorum
Source: Front Vet Sci. 2020 Nov 5;7:571361. doi: 10.3389/fvets.2020.571361 (PMC7674402; doi:10.3389/fvets.2020.571361)
Supplement: Supplementary file 1 [file Data_Sheet_1.docx]

Supplementary Material

# Supplementary Tables and Figures

## Supplementary Tables

**Table S1. Primers used in this study.**

| Primer name | Sequences of primer (5'-3') | Purpose |
| --- | --- | --- |
| rpoS-F | ATGAGTCAGAATACGCTGAAAGTTC | Cloning of *rpoS* |
| rpoS-R | TTACTCGCGGAACAGCGCTTCGATA | Cloning of *rpoS* |
| Q-gyrB-F | ACGCGTCTGTTGACCTTCTTC | Quantitative real-time PCR |
| Q-gyrB-R | CTGTTCCTGCTTACCTTTCTTCAC | Quantitative real-time PCR |
| Q-csgD-F | CGGCCGGTTGCATTGTTTTA | Quantitative real-time PCR |
| Q-csgD-R | CCACGTGTTCCTGGTCTTCA | Quantitative real-time PCR |
| Q-csgA-F | TCGACCAGTGGAACGCTAAAA | Quantitative real-time PCR |
| Q-csgA-R | ACCAACCTGACGCACCATTAC | Quantitative real-time PCR |
| Q-bcsA-F | CGGGCGTGAATCATTTCGTC | Quantitative real-time PCR |
| Q-bcsA-R | TCAGGAACCAGCCCATTGTC | Quantitative real-time PCR |
| Q-rpoS-F | CGTCAGCCGTATGCTTCG | Quantitative real-time PCR |
| Q-rpoS-R | CGCTGTTTGGCGTTCAGTT | Quantitative real-time PCR |
| rpoS-yF1 | GGATCCATGAGTCAGAATACGCT | Construction of pET32a-7S and pET32a-9S |
| rpoS-yR1 | CTCGAGTTACTCGCGGAACAG |  |
| rpoS-HF | CGGGATCCATGAGTCAGAATACGCTGAAAGTTC | Construction of pGEX-6P-1-7S and pGEX-6P-1-9S |
| rpoS-HR | CCGCTCGAGTTACTCGCGGAACAGCGCTTCGATA |  |
| PGEX-F | GGATCCCCGGAATTCCCG | Construction of pGEX-6P-1-p7 and pGEX-6P-1-p9 |
| PGEX-R | ACAGCTCATTTCAGAATATTTGCCA |  |
| PrpoS-F | aatattctgaaatgagctgtgaattcTTCTGAGGGCTCAGGTGAACA |  |
| PrpoS-R | cccgggaattccggggatccTTACTCGCGGAACAGCGC |  |
| csgD-F | GGTACCCTGTCCAGGTTAATGC | Construction of pcsgD-lacZ |
| csgD-R | GGATCCGTTTTTGCGGCTTA | Construction of pcsgD-lacZ |
| lacZ-F | CGGGATCCAAGACCAGAAACAGCA | Construction of pcsgD-lacZ |
| lacZ-R | TGCACTGCAGCAAACCACAACTAGAATG | Construction of pcsgD-lacZ |
| PcsgD-F | TGCCTGCAGGTCGACGATGCAATAACAGCGAAATGTACAAC | Amplify promotor of *csgD* with FAM probe |
| PcsgD-R | GCTGTGGGTTGAAATAGCCC |  |
| FAM | TGCCTGCAGGTCGACGAT |  |
| 193-9B-F | ACCACGAACTGAGTGCGGAAGAAATTGCAGAGCAA | Construction of pGEX-6P-1-9B |
| 193-9B-R | CTTCCGCACTCAGTTCGTGGTCCAGTTTATGCGAC | Construction of pGEX-6P-1-9B |
| 193-7B-F | GACCACGAACCGAGTGCGGAAGAAATTGCAGAGCA | Construction of pGEX-6P-1-7B |
| 193-7B-R | TCTTCCGCACTCGGTTCGTGGTCCAGTTTATGCGA | Construction of pGEX-6P-1-7B |
| rpoS-cat-F | AGGCTTTGACTTGCTAGTTCCGTCAAGGGATCACGGGTAGGAGCCACCTTATGAGTCAGAATACGCTGAAAGTTC | Cloning of *rpoS-cat* |
| rpoS-cat-R | GGTAAAAAAAAGGCCAGTCTGTCGACTGGCCTTTTTTTGACAAGGGTACCATATGAATATCCTCCTTAGTTCCT | Cloning of *rpoS-cat* |
| rpoS-F1 | ATGAGTCAGAATACGCTGAAAGTTC | Cloning of *rpoS* |
| rpoS-R1 | ACTTCGAAGCAGCTCCAGCCTACACTTACTCGCGGAACAGCGCTT | Cloning of *rpoS* |
| cat-F1 | GTGTAGGCTGGAGCTGCTTCGAAGT | Cloning of *cat* gene |
| cat-R1 | CATATGAATATCCTCCTTAGTTCCT | Cloning of *cat* gene |

## Supplementary Figures

**
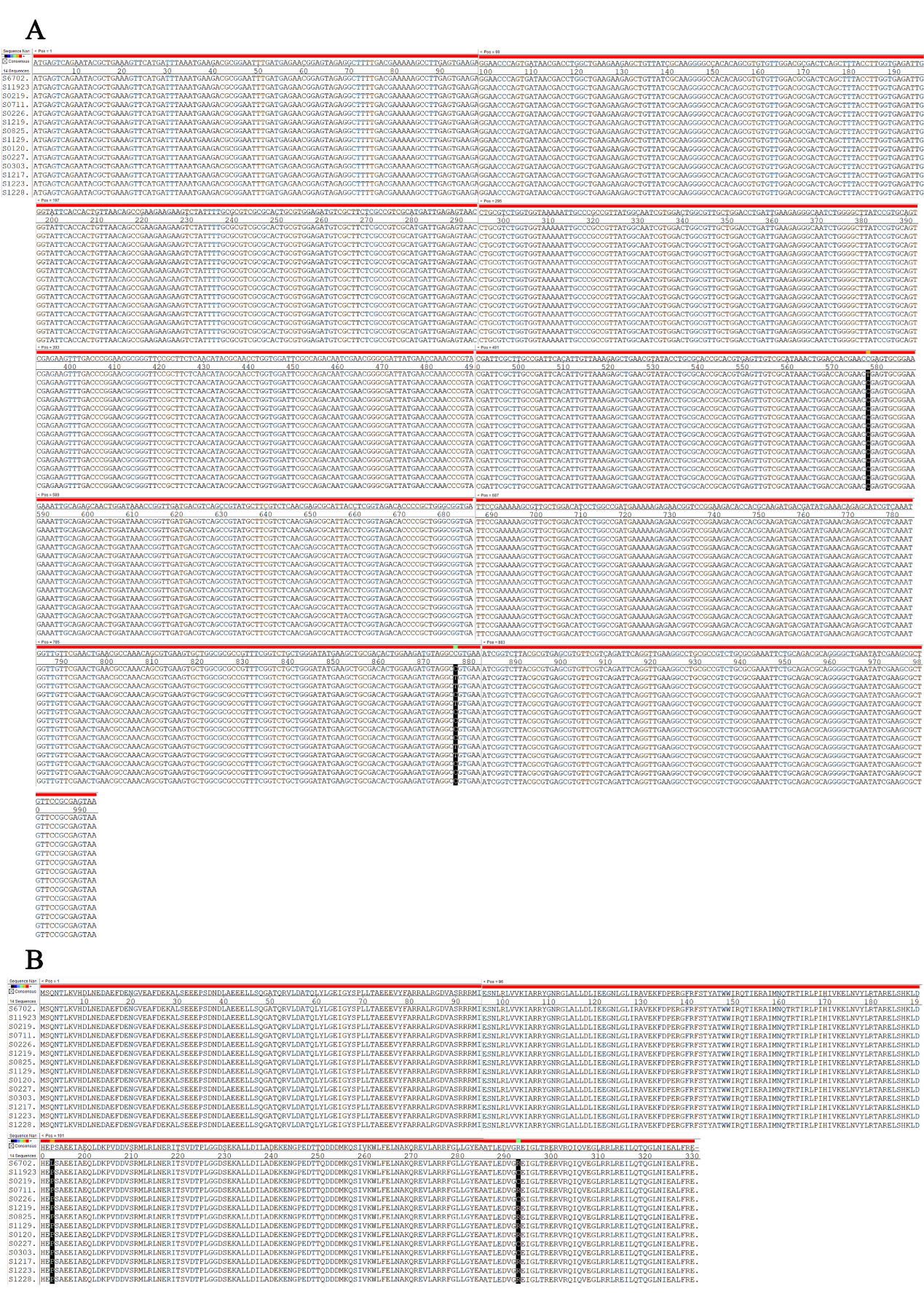
**

**Figure S1. Nucleotide alignment (A) and amino acid alignment (B) of *rpoS* genes.**


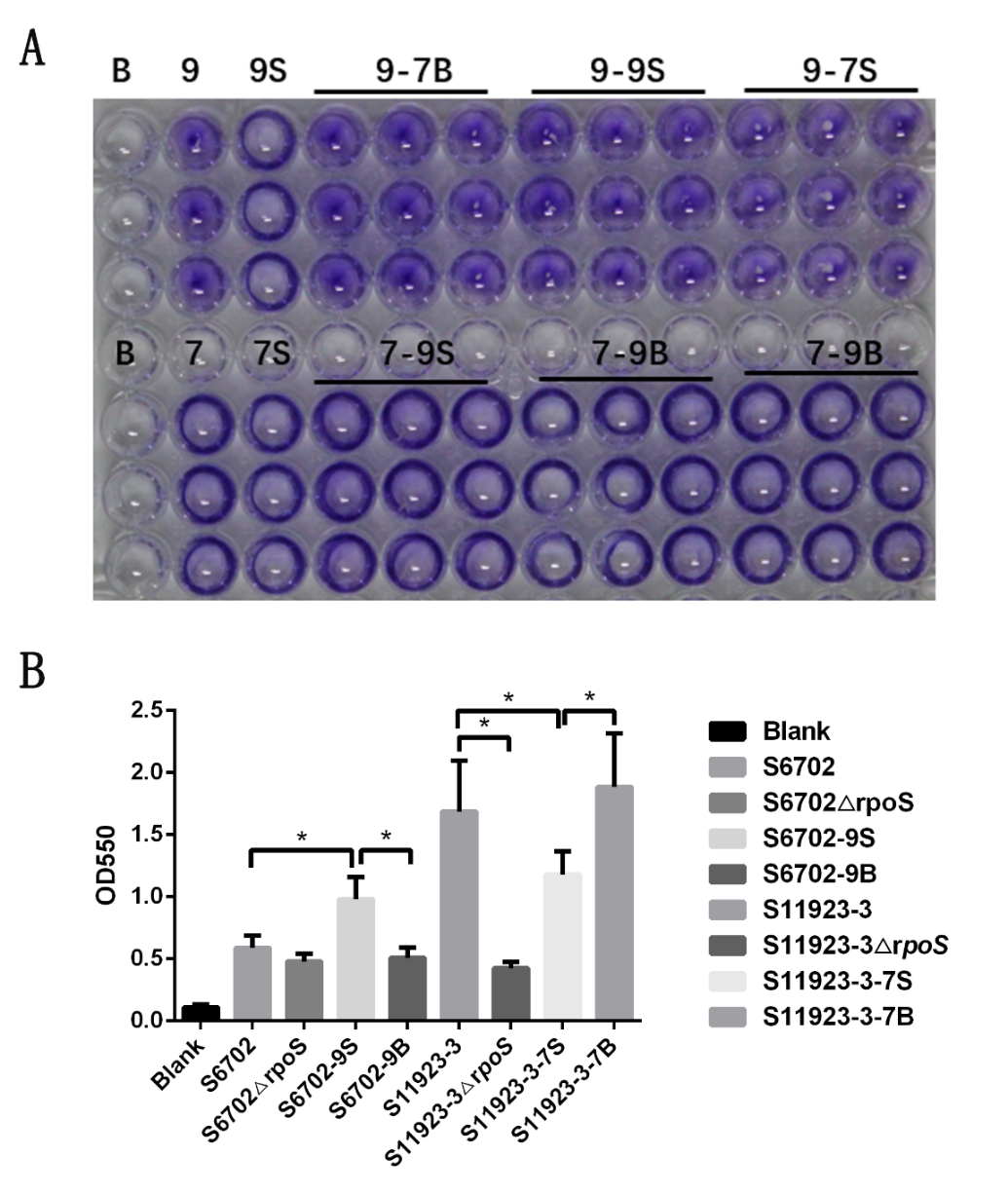


**Figure S2.** **Determination of biofilm formation of S6702 and S11923-3 with residues 193 and 293 substitution in RpoS. (A)** All of the strains were cultured in TSB medium first. After overnight cultivation, cultures were diluted in 1/10 TSB and grown in 96-well plates (100μL/ well) at 28°C for 24 h without shaking. Then the supernatant was discarded and the wells were washed gently with distilled water to remove non-adherent bacteria. The plates were stained with 0.4% crystal violet. **(B)** The crystal violet was solubilized with 100 μL of 25% acetone with anhydrous ethanol. Crystal violet staining quantification was tested by measuring the optical density (OD_550_). Means and standard deviations from three independent experiments are shown.


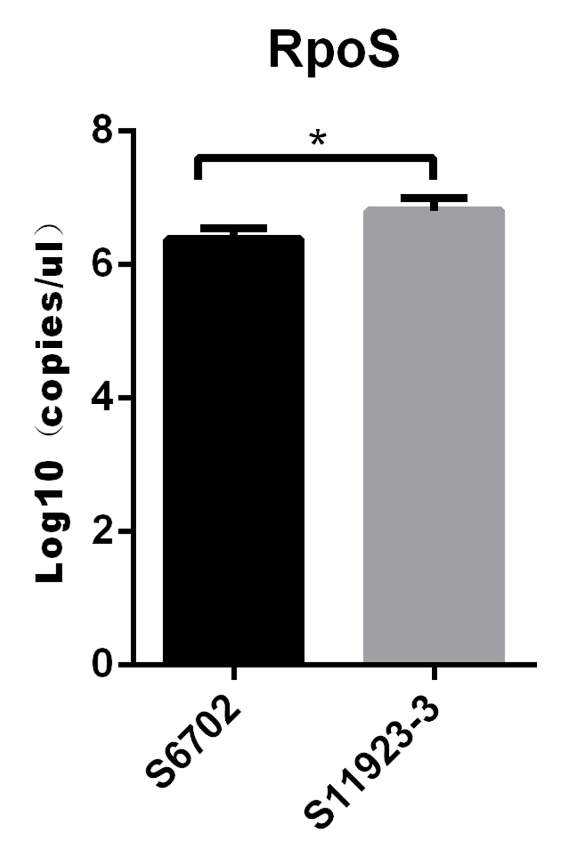
**Figure S3.** **Determination of RpoS transcript levels of S6702 and S11923-3.** S6702 and S11923-3 were cultured in TSB medium overnight and then diluted in 1/10 TSB medium in small dishes at 28℃ without shaking for 24 h. The total RNAs of scraped samples were extracted using a Bacterial RNA Kit (Omega). The cDNA was synthesized using a PrimeScript RT reagent Kit with gDNA Eraser (Takara) and quantified via TB Green Premix Ex Taq (Takara). The *rpoS* transcript levels in these strains were tested by absolute quantification using primer pair Q-rpoS-F/R and the formula for the standard curve was Y= -3.4X+37.6.
